# A Low-Modulus Phosphatidylserine-Exposing Microvesicle Alleviates Skin Inflammation via Persistent Blockade of M1 Macrophage Polarization

**DOI:** 10.3390/ijms26010394

**Published:** 2025-01-04

**Authors:** Zihao Zhang, Yidi Mo, Shengxia Xu, Lei Jiang, Yuanshu Peng, Yani ZhuGe, Zhijian Su, Qi Xiang, Rong Zeng, Guanglin Zhang

**Affiliations:** 1 Guangdong Provincial Key Laboratory of Bioengineering Medicine, Department of Cell Biology, Jinan University, Guangzhou 510632, China; a9343371462022@163.com (Z.Z.); moyidi2022@163.com (Y.M.); xshengx1130@163.com (S.X.); 18279333543@163.com (L.J.); zhugeyani010413@163.com (Y.Z.); tjnuszj@jnu.edu.cn (Z.S.); txiangqi@jnu.edu.cn (Q.X.); 2 Department of Material Science and Engineering, College of Chemistry and Materials Science, Jinan University, Guangzhou 510632, China; pengys02@163.com; 3 College of Biology and Agriculture, Shaoguan University, Shaoguan 512005, China

**Keywords:** inflammatory skin diseases, microvesicle, low modulus, phosphatidylserine, therapy

## Abstract

Inflammatory skin diseases comprise a group of skin conditions characterized by damage to skin function due to overactive immune responses. These disorders not only impair the barrier function of the skin but also deteriorate the quality of life and increase the risk of psychiatric issues. Here, a low-modulus phosphatidylserine-exposing microvesicle (deformed PSV, D-PSV) was produced, characterized, and evaluated for its potential therapeutic function against skin diseases. Compared to conventional PSVs (C-PSVs), D-PSVs exhibited a more robust and longer-lasting inhibitory effect on the inflammatory response triggered by lipopolysaccharides and interferon-γ in a primary bone marrow-derived macrophage model. Transcriptome analysis indicated that the inhibitory effect of D-PSVs was mainly achieved by modulating inflammation-related signaling pathways, leading to a reduction in the expressions of pro-inflammatory genes. In an imiquimod-induced psoriatic dermatitis mouse model, topical application of D-PSVs effectively mitigated inflammation in the skin microenvironment and reduced lesion severity. These improvements were attributed to the superior skin permeability and more persistent adhesion of D-PSVs to macrophages compared with C-PSVs. In summary, this macrophage-targeted microvesicle offers a promising non-invasive approach to managing inflammatory skin diseases by persistently inhibiting M1 macrophage polarization and restoring immune microenvironment balance.

## 1. Introduction

Inflammatory skin conditions such as eczema, dermatitis, psoriasis, acne, and allergic urticaria affect people of all ages, genders, and races [1]. These diseases are characterized by immune system disorders, abnormal skin barrier function, a non-contagious nature, frequent recurrence, and complex etiology [2]. Until now, the exact causes and mechanisms of many inflammatory skin diseases still remain unclear, leading to a lack of targeted therapeutic treatments.

Clinically, these conditions present with symptoms such as itching, blisters, redness, swelling, crusting, and scaling [3]. Common treatments involve the topical application of corticosteroids or phosphatase inhibitors to alleviate symptoms [4,5]. While effective in the short term, long-term use of these medications can weaken the immune system and pose risks such as adrenal suppression, growth retardation, hypertension, hyperglycemia, insulin resistance, cataracts, and even malignancies [6].

Immune system cytokines are crucial in facilitating communication between immune and non-immune cells. Inflammatory factors like tumor necrosis factor-alpha (TNF-α), interleukin 1 (IL-1), interleukin 6 (IL-6), and interleukin 12 (IL-12), primarily secreted by macrophages and dendritic cells, activate host defenses and maintain immune balance by initiating inflammation [7]. However, abnormal spatiotemporal expression of cytokine can lead to dysfunctional immune responses and immune-related skin diseases. More importantly, persistent accumulation of inflammatory factors in the skin epithelial microenvironment can disrupt the balance of feedback mechanisms in immune regulation and make the disease difficult to cure [8]. Therefore, reducing the concentration of pro-inflammatory factors in the microenvironment is crucial for the alleviation and treatment of inflammatory skin diseases.

Apoptotic cell-derived extracellular microvesicles (ApoMVs) (<1000 nm in diameter) produced during the progression of apoptosis play an important role in immune regulation [9,10,11]. It has been well established that smaller ApoMVs can expose the “eat-me” signal of phosphatidylserine (PS) on their surface like apoptotic cells to trigger more efficient recognition and engulfment by macrophages via phagocytic receptors such as CD36 than the whole apoptotic cells and signal macrophages to adopt an anti-inflammatory phenotype [12,13]. Synthetic ApoMVs, including PS-exposing polymeric nanoparticles and liposomes, can replicate the anti-inflammatory and immunosuppressive properties of natural ApoMVs and offer innovative therapeutic strategies for inflammatory diseases like myocarditis, hepatic inflammation, and retinal injury by modulating macrophage functions [14,15,16,17,18,19,20]. Interestingly, our previous studies indicated that Young’s modulus plays a critical role in regulating the interaction between ApoMVs-inspired PS-exposing microvesicles (PSVs) and macrophages [21,22]. However, the precise mechanisms underlying these interactions and their implications for treating skin inflammation remain underexplored.

In this study, we constructed PSVs with a distinct Young’s modulus and systematically evaluated their anti-inflammatory effects using primary macrophage models and transcriptomic analysis. Furthermore, we assessed the therapeutic efficacy of these PSVs in a murine model of imiquimod-induced psoriasis, a well-established experimental platform for studying inflammatory skin diseases. Finally, we investigated the underlying mechanisms contributing to the observed anti-inflammatory outcomes, providing insights into the role of mechanical properties in PSV-mediated immunomodulation.

## 2. Results

### 2.1. Fabrication and Characterization of PSVs

**Figure 1:**
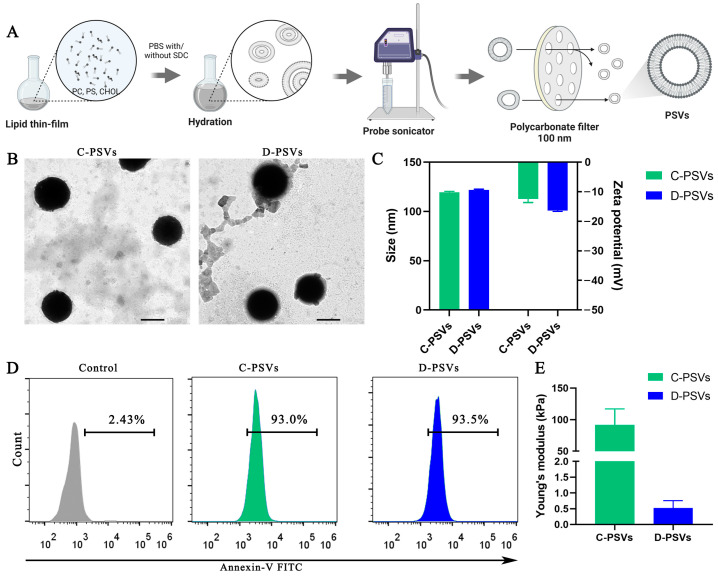
Characterization of C-PSVs and D-PSVs. (**A**) Schematic representation of PSV preparation. (**B**) TEM images depicting the morphology of C-PSVs and D-PSVs. Scale bar = 200 nm. (**C**) Hydrodynamic diameter (size) and zeta potential measurements of C-PSVs and D-PSVs. (**D**) Flow cytometry analysis of PS exposure on the surface of PSVs, with microvesicles lacking PS used as a negative control. PS exposure was quantified by the specific binding of Annexin V. The data are presented as the percentage of Annexin V-positive PSVs. (**E**) AFM analysis of the Young’s modulus of C-PSVs and D-PSVs. Data are represented as mean ± S.E.M.

C-PSVs and D-PSVs with identical PS content but a distinct Young’s modulus were prepared using DOPC, DPPS, cholesterol, and SDC via the thin-film hydration and extrusion method (Figure 1A) [23]. Transmission electron microscopy (TEM) revealed that both types of PSVs had a spherical morphology with diameters of approximately 100 nm (Figure 1B). Dynamic light scattering (DLS) and zeta potential measurements confirmed a similar particle size of around 120 nm for both PSVs, consistent with TEM observations, and indicated negative surface charges (Figure 1C).

Flow cytometry analysis was employed using the specific recognition of PS by Annexin-V staining [24] to verify the presence of PS on the vesicle surface. Compared to microvesicles lacking PS, over 90% of the Annexin V-positive events originated from both PSVs, thereby confirming the successful incorporation of PS onto their surface (Figure 1D). Moreover, atomic force microscopy (AFM) combined with the Hertz model demonstrated that C-PSVs exhibited a significantly higher Young’s modulus (91 ± 26 kPa) compared to D-PSVs (0.5 ± 0.2 kPa), confirming that the introduction of SDC into the lipid bilayer resulted in the formation of more deformable PSVs (Figure 1E).

### 2.2. PSVs Have the Capacity to Inhibit Macrophage Inflammatory Response In Vitro

**Figure 2:**
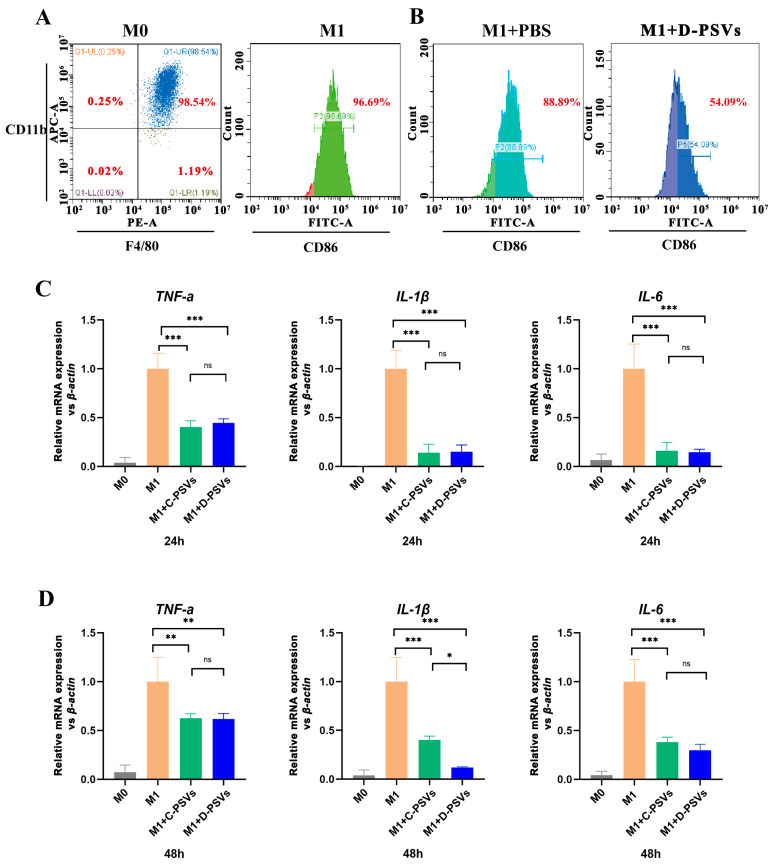
In vitro assessment of PSV impact on M1-type macrophages. (**A**) Flow cytometry analysis of BMDMs expressing CD11b^+^ and F4/80^+^ markers. (**B**) The effect of D-PSVs on CD86 expression of LPS- and IFN-γ-induced macrophages. (**C**,**D**) PSVs were co-incubated with M1-type macrophages for 24 and 48 h, followed by quantitative qRT-PCR analysis of inflammatory gene expression. ns indicates no significant difference; * *p <* 0.05, ** *p <* 0.01, *** *p <* 0.001.

Primary BMDMs were isolated from adult male C57BL/6 mice. Flow cytometry analysis showed that more than 98.5% of isolated cells expressed the surface markers CD11b^+^ and F4/80^+^ (Figure 2A). The BMDMs were stimulated with LPS and IFN-γ for 24 h to establish a standard M1 macrophage model, leading to a significant increase in CD86 expression, a marker commonly associated with M1 polarization (Figure 2A). These M1 macrophages were then treated with either a D-PSV solution at a lipid concentration of 0.5 μmol/mL or PBS for 24 h. It was clear that D-PSV treatment significantly reduced the ratio of cells expressing CD86 compared to the PBS group (Figure 2B).

To further assess the impact of PSVs on LPS- and IFN-γ-induced M1 macrophage polarization, the BMDMs were co-cultured with either C-PSVs or D-PSVs in the presence of M1-inducing agents for either 24 or 48 h. The qRT-PCR results indicated that LPS and IFN-γ stimulation significantly elevated the mRNA expressions of several featured pro-inflammatory cytokines, including *Tnf-α*, *Il-1β*, and *Il-6*. Both C-PSV and D-PSV treatments could decrease the mRNA expressions of these cytokines during the induction (Figure 2C,D), while D-PSVs exhibited a more robust and longer-lasting inhibitory effect on *Il-1β* expression. Notably, the surfactant SDC showed no inhibitory effect on the expressions of *Tnf-α* and *Il-1β* in M1 macrophages, suggesting that the anti-inflammatory effect of D-PSVs is independent of the SDC component (Supplemental Figure S1).

Taken together, these findings suggested that PSVs could effectively attenuate the LPS- and IFN-γ-induced inflammatory response in BMDMs, which were mediated by the PS on the vesicle surface [25].

### 2.3. The Transcriptomic Analysis of PSV-Inhibited M1 Macrophage Polarization

BMDMs were polarized with either IFN-γ and LPS or IL-4 to differentiate into M1 or M2 macrophages, respectively, to evaluate the function of PSVs in regulating the fate of macrophages. These M0, M1, and M2 macrophages exhibited specific surface markers characteristic of their phenotypes. The M1 macrophages were further treated with C-PSVs or D-PSVs in the presence of M1-inducing agents for 24 h.

All cells were harvested, and RNA sequencing was conducted. Differentially expressed genes (DEGs) were identified using a threshold of a minimum 2-fold change in expression and a false discovery rate (FDR) of less than 0.05. Transcriptomes from M0, M1, M2, M1_C (C-PSV-treated M1 macrophages), and M1_D (D-PSV-treated M1 macrophages) groups were analyzed, detecting around 24,395 genes and identifying 5717 DEGs (Supplemental Table S2). Spearman’s correlation was used to analyze transcriptomic similarities and differences among the five groups. Results indicated that M1 and M0 macrophages had a correlation coefficient of 0.88–0.92, which was similar to the coefficient between M2 and M0 (0.87–0.89), while the correlation between M1 and M2 was lower at 0.81–0.83 (Supplemental Figure S2). There was no significant difference between the global gene expression patterns of D-PSV- and C-PSV-treated groups, although D-PSV treatment appeared to shift M1 macrophages more towards an M2-like macrophage phenotype.

**Figure 3:**
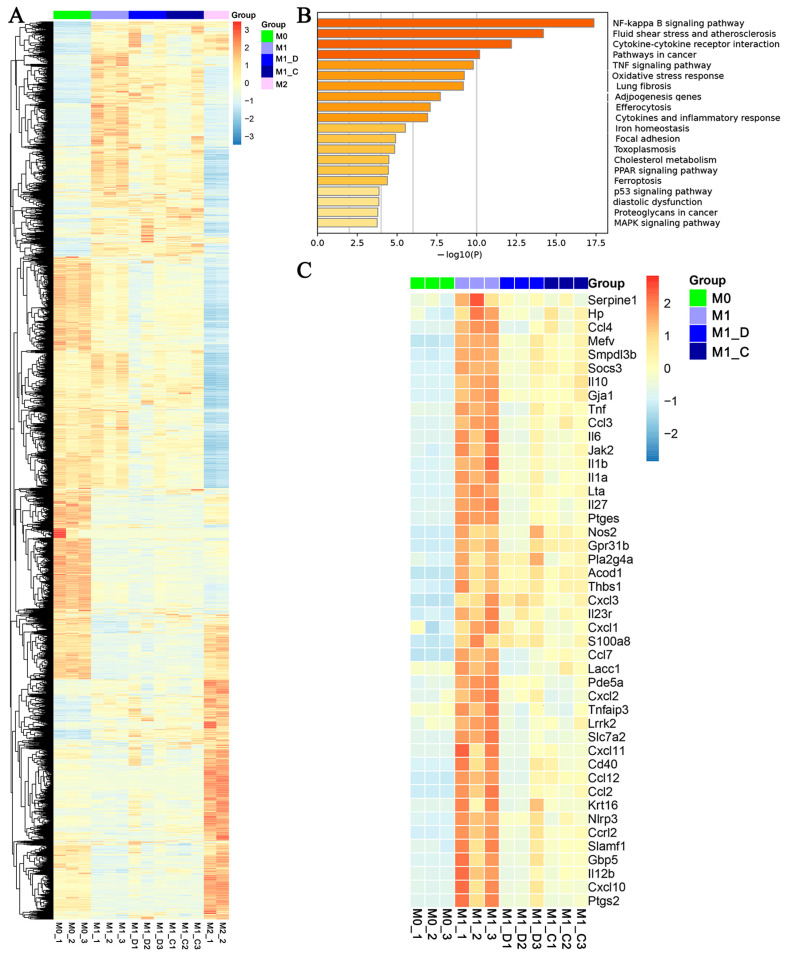
PSVs suppress M1 macrophage polarization and inflammatory response. (**A**) Heatmap of differentially expressed genes in M0, M1, M2, and M1 macrophages treated with C-PSVs and D-PSVs. (**B**) KEGG pathway analysis of DEGs from D-PSV- and C-PSV-treated M1 macrophages. (**C**) Heatmap analysis of immune-related DEGs from M0s, M1s, D-PSV-treated M1 macrophages, and C-PSV-treated M1 macrophages.

Based on the in vitro results, we next investigated potential changes in PSV treatment. Sequencing data revealed that 661 and 789 genes had remarkably changed expression levels in the D-PSV and C-PSV groups, respectively, compared to the M1 macrophage group after 24-h treatment (Figure 3A). KEGG analysis showed significant enrichment in pathways such as NF-κB, TNF, PPAR, p53, and MAPK signaling (Figure 3B). Further screening of inflammatory-related genes indicated that both C-PSVs and D-PSVs could downregulate key pro-inflammatory mediators, including *Tnf-α*, *Il-1α*, *Il-1β*, *Il-6*, and *Il-12*, as well as chemokines such as *Ccl-2*, *Cxcl-3*, *Ccl-3*, *Ccl-4*, *Ccl-7*, and *Ccl-12* (Figure 3C).

Taken together, both C-PSVs and D-PSVs can suppress M1 macrophage polarization by decreasing the expressions of key pro-inflammatory factors, highlighting their potential in modulating inflammation-related pathways.

### 2.4. D-PSVs Ameliorate Skin Lesions in an IMQ-Induced Psoriatic Dermatitis Mouse Model

**Figure 4:**
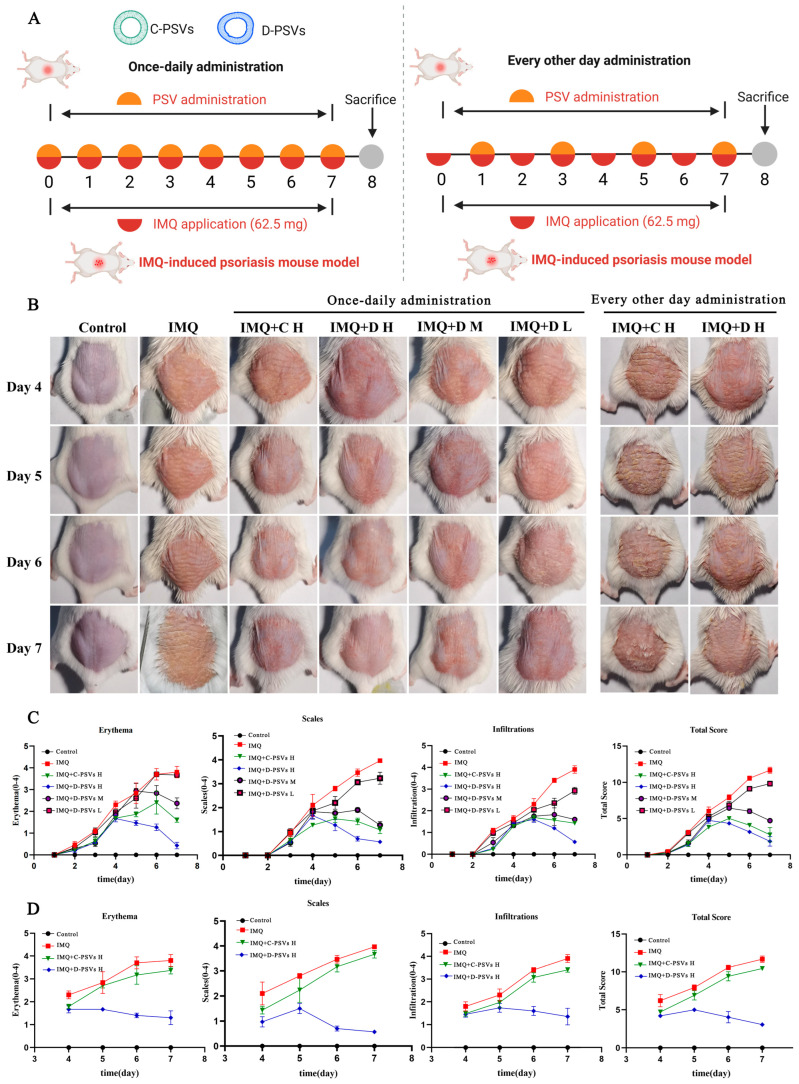
PSVs ameliorate IMQ-induced psoriasiform skin lesions in mice. (**A**) Therapeutic regimens of PSVs in IMQ-induced psoriasis models: daily versus alternate-day administration. (**B**) Representative lesion images on days 4 to 8. (**C**) The quantified analysis of the Psoriasis Area and Severity Index (PASI) scores. (**D**) PASI scores for alternate-day dosing regimens.

To evaluate the in vivo therapeutic efficacy of PSVs, we established an IMQ-induced psoriasiform dermatitis model in mice and subsequently treated the mice with PSVs (Figure 4A). It is well-documented that this model replicates key features of human psoriasis, including erythema, scaling, and immune cell infiltration [26]. The mice received topical applications of 5% IMQ cream daily on their shaved dorsal skin for four consecutive days, successfully inducing visible psoriasiform dermatitis characterized by erythema and scaling. Compared to normal mice, the IMQ-treated animals exhibited significantly elevated Psoriasis Area and Severity Index (PASI) scores.

According to the instructions described in the materials Section 4.7, we topically applied different doses (0.07, 0.23, and 0.7 μmol) of D-PSVs and a high dose of C-PSVs (0.7 μmol) in 70 μL of PBS to the psoriatic lesions daily. The results indicated that both PSV treatments significantly reduced the psoriatic area compared to the model group, and the therapeutic effect of D-PSVs was in a dose-dependent manner (Figure 4B). Moreover, the high-dose D-PSV application displayed more efficiency in reducing the psoriatic area than that of the high-dose C-PSVs, resulting in significantly diminished erythema, skin thickening, immune cell infiltration, and total PASI scores at the treatment endpoint (Figure 4C).

Splenomegaly is a hallmark signature of ongoing inflammatory processes. This situation was clearly observed in the IMQ-induced model group and confirmed by a significantly elevated spleen index (spleen weight/body weight ratio) on days 5 and 7 (Supplementary Figure S3). The high-dose D-PSVs displayed the most optimal suppression of inflammation in the animals, with the spleen index approaching that of the normal group.

To evaluate the sustainability of PSV treatments, we investigated the efficacy of alternate-day dosing regimens. The morphological observations revealed an improvement in the psoriatic phenotype in the high-dose D-PSV treatment compared to the C-PSV group, characterized by a significant reduction in the psoriatic lesion area (Figure 4A). Furthermore, the PASI scores at the treatment endpoint with D-PSVs were also significantly lower than those of C-PSVs (Figure 4D).

These findings suggest that D-PSVs exert a more effective and sustained inhibitory effect on psoriasis-like skin inflammation.

### 2.5. D-PSVs Exert Enhanced Anti-Inflammatory Effects in Skin Microenvironments

**Figure 5:**
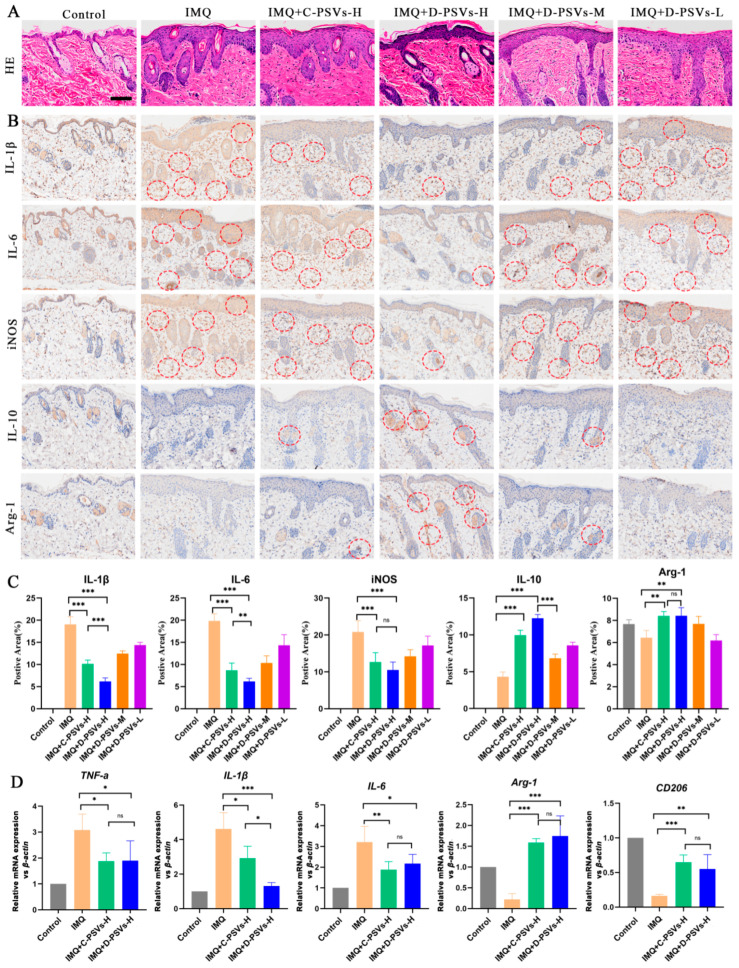
The assessment of PSV anti-inflammatory responses in psoriatic skin tissue. (**A**) Histopathological analysis of mouse dorsal skin on day 7 post-treatment using H&E staining. Scale bar = 100 μm. (**B**) Immunohistochemical (IHC) analysis of M1 and M2 macrophage marker expressions in psoriatic mouse skin treated with C-PSVs or D-PSVs on day 7. Representative images display the expression levels of pro-inflammatory markers IL-1β, IL-6, and iNOS (M1 phenotypes) and anti-inflammatory markers Arg-1 and IL-10 (M2 phenotypes). Positive expressions are indicated by red circles. (**C**) Quantification of IHC staining intensity for M1 and M2 markers in panel (**B**) using ImageJ software. The intensity measurements provide a comparative analysis of marker expression between treatment groups. (**D**) The qRT-PCR analysis of mRNA expression levels for pro-inflammatory cytokines (IL-1β, IL-6, TNF-α) and M2 markers (CD206, Arg-1) in psoriatic skin lesions. ns indicates no significant difference; * *p <* 0.05, ** *p <* 0.01, *** *p <* 0.001.

Histopathological analyses investigated the changes in skin treated with PSVs in the IMQ-induced psoriasiform mouse model (Figure 5A). Compared to the normal control, IMQ treatment induced substantial epidermal and dermal thickening, along with pronounced elongation of the rete ridges. D-PSV treatment significantly reduced epidermal and dermal thickening and restored the rete ridges to a normalized morphology. Although high-dose C-PSVs also exhibited similar beneficial effects, residual thickening suggested limited efficacy compared to high-dose D-PSVs. Furthermore, this improvement of D-PSVs in histopathological parameters, together with the damage scores and skin observations, strongly supports the efficacy of D-PSVs in alleviating skin symptoms of psoriasis.

Abnormal expressions of multiple pro-inflammatory cytokines are key contributors to psoriatic onset and progression [27]. Immunohistochemical (IHC) analysis indicated significantly elevated levels of IL-1β, IL-6, and inducible nitric oxide synthase (iNOS) in the IMQ-induced psoriatic lesions. Treatments with C-PSVs or D-PSVs were able to significantly downregulate the expressions of pro-inflammatory genes, with D-PSVs exhibiting a pronounced effect in a dose-dependent manner (Figure 5B,C). Importantly, the IMQ-induced downregulation of the anti-inflammatory cytokine IL-10 and the anti-inflammatory macrophage marker arginase-1 (ARG-1) was recovered by PSVs, especially high-dose D-PSVs. The in vitro experiments using the LPS-induced M1 macrophage polarization model further demonstrated that D-PSVs exhibited a greater capacity to promote CD206 upregulation compared to C-PSVs at 48-h treatment (Supplementary Figure S4), suggesting a potential to facilitate the phenotypic switch of M1 to the M2-like macrophage phenotype.

The qRT-PCR results also clearly indicated that the mRNA expression levels of *Tnf-α*, *Il-1β*, and *Il-6* were significantly elevated in the dorsal skin of model mice (Figure 5D), accompanied by a notable decrease in the anti-inflammatory macrophage markers ARG-1 and CD206. Both high-dose D-PSVs and C-PSVs could efficiently inhibit the expressions of inflammatory cytokines and promote the expressions of M2 macrophage marker genes.

In short, these findings suggest that D-PSVs have a more pronounced therapeutic effect in the IMQ-induced psoriatic mouse model and a sustained potential to improve the skin microenvironment.

### 2.6. D-PSVs Have Excellent Skin Permeability

**Figure 6:**
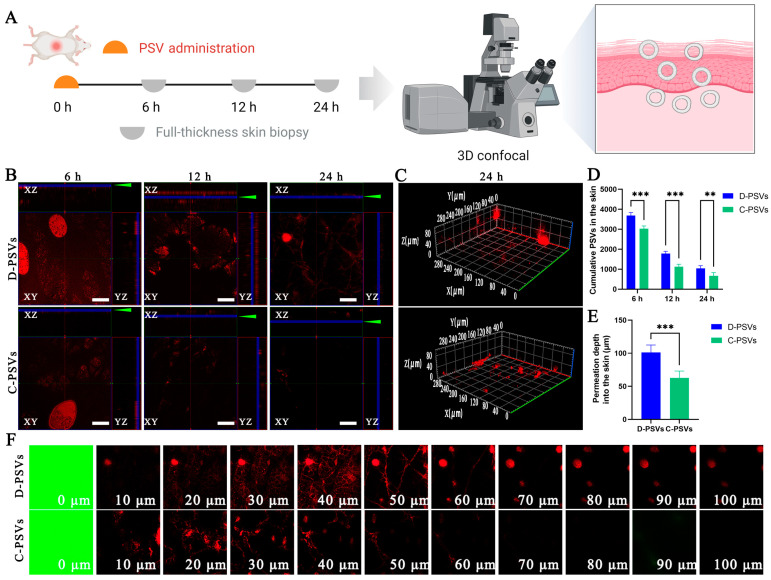
The permeation assessment of PSVs in mouse skin. (**A**) DID-labeled PSVs were topically applied to the shaved back skin of mice and detected at 6, 12, and 24 h post-application. (**B**) Distribution of PSVs on mouse skin. (**C**) Cross-sectional CLSM imaging at 24 h showing the depth of PSV penetration into the skin layers. (**D**) Quantification of transdermal permeation and accumulation of C-PSVs and D-PSVs in intact mouse skin. (**E**,**F**) Penetration depth of PSVs. The scale bars represent 50 μm. ** *p <* 0.01, *** *p <* 0.001.

To understand the differences between C-PSVs and D-PSVs in their therapeutic effects on skin inflammatory diseases, we hypothesized that their ability to penetrate the skin was different. DID-labeled vesicle solutions of both types were applied to the shaved dorsal skin of healthy mice. After 6-, 12-, and 24-h treatments, whole skin samples were collected for three-dimensional (3D) confocal laser scanning microscopy (CLSM) analysis (Figure 6A). Compared with C-PSVs, the DID signal intensity of D-PSVs in the deeper skin layers showed stronger and faster accumulation over time (Figure 6B). Quantitative analysis revealed that the cumulative skin retention of D-PSVs was significantly higher than that of C-PSVs, with 1.22-, 1.58-, and 1.56-fold increases at 6, 12, and 24 h post-administration, respectively (Figure 6D). Cross-sectional 3D reconstruction results also confirmed a greater accumulation of D-PSVs in the deeper skin layers (Figure 6C). Additionally, CLSM analysis using FITC-labeled vesicles showed that the average detectable fluorescence penetration depth of the D-PSVs exceeded 100 µm, while that of the C-PSVs was less than 70 µm (Figure 6E,F).

### 2.7. D-PSVs Exhibit Persistent Adherence to the Macrophage Surface and Suppress M1 Polarization

**Figure 7:**
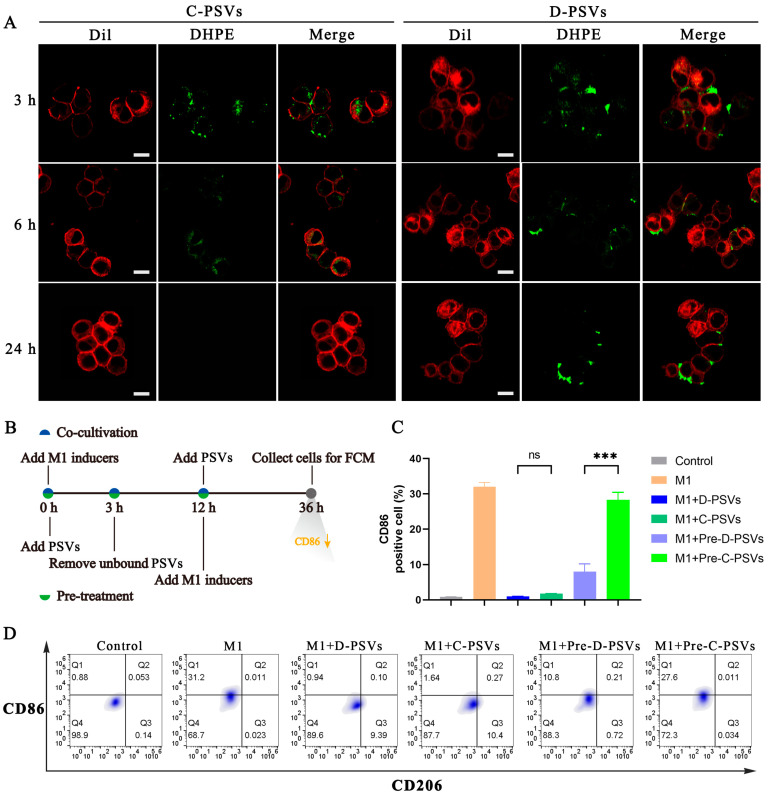
D-PSVs exhibit persistent surface attachment to macrophages and suppress M1 polarization. (**A**) Confocal microscopy analysis of PSV internalization. Macrophages were co-cultured with PSVs for 3 h, followed by extensive washing to remove unbound PSVs. Cells were then incubated for an additional 3, 6, and 24 h before fixation and staining. Red indicates the cell membrane (Dil probe), and green indicates PSVs (DHPE probe). Scale bar = 20 μm. (**B**) Experimental timeline detailing the co-culture and pre-treatment conditions. (**C**,**D**) Flow cytometry analysis of M1 and M2 macrophage marker expression. BMDMs were incubated with PSVs according to the protocol detailed in (**B**). Cells were harvested and stained for CD86 (M1 marker) and CD206 (M2 marker) expression, followed by flow cytometry analysis. ns represents no significant difference; *** *p* < 0.001.

Phosphatidylserine exposed on the surface of apoptotic cells is recognized by macrophages via receptors that can bind PS either directly or indirectly, triggering a complex array of cytoskeletal rearrangements to facilitate the engulfment of foreign matter [28,29]. Consequently, we propose that the duration of interaction between PSVs and the macrophage membrane is a critical determinant of their therapeutic efficacy. To investigate the interaction dynamics between PSVs and macrophages, we monitored their co-localization over time. Fluorescence microscopy observed that both C-PSVs and D-PSVs were rapidly recognized and bound by macrophages within 3 h, primarily associated with the cell surface (Figure 7A). After 6 h, D-PSVs exhibited distinct behavior compared to C-PSVs. While C-PSVs were predominantly internalized, D-PSVs showed remarkable resistance to internalization, remaining predominantly attached to the cell membrane. This persistent adherence was further confirmed at 24 h, with C-PSVs showing minimal fluorescence intensity due to potential lysosomal degradation, whereas D-PSVs remained largely bound to the cell surface.

To elucidate the impact of macrophage-associated C-PSVs and D-PSVs on M1 macrophage polarization, we evaluated the expressions of M1 marker CD86 and M2 marker CD206 following co-cultivation or pre-treatment with PSVs (Figure 7B). Flow cytometry analyses revealed a significant increase in CD86 expression upon exposure to M1-polarized inducers, with no effect on CD206. Interestingly, the presence of either C-PSVs or D-PSVs significantly inhibited CD86 expression (Figure 7C,D), with both groups exhibiting a similar suppressive effect, approaching the level of the control group. These findings are consistent with our previous observations of PSV-mediated inhibition of inflammatory gene expressions in primary macrophages.

The protective effect of pre-treated C-PSVs against M1 macrophage polarization was quickly diminished after the removal of free C-PSVs. In contrast, pre-treated D-PSVs, due to their persistent adhesion to the macrophage surface, displayed a sustained inhibitory effect on M1 polarization. In addition, the presence of LPS and IFN-γ in the microenvironment is essential for maintaining M1 polarization. Removal of LPS and IFN-γ led to a significant decline in M1 polarization markers, including a substantial downregulation of *Tnf-α* and *Il-1β* expression (Supplementary Figure S5). These results suggest that the high internalization resistance and persistent cell membrane association of D-PSVs effectively block the continuous stimulation of the M1-polarized inducers, mimicking a reduction in LPS and IFN-γ stimulation and leading to a more pronounced suppression of M1 polarization.

## 3. Discussion

Cell apoptosis and its phagocytic clearance represent an innate immune silencing mechanism that has inspired the design of anti-inflammatory nanoparticles [30,31]. These nanoparticles integrate the apoptotic signal PS onto biomaterial surfaces to target macrophages and elicit anti-inflammatory responses [32]. For example, the Kraynak group successfully modulated inflammatory macrophages using PS-modified 3T3 cell membrane-coated poly (lactide-co-glycolide) (PLGA) nanoparticles [33]. Similarly, Zhang and colleagues proposed a novel oxidative stress-responsive self-delivery method that mimics phagocytosis, replicating the anti-inflammatory and pro-repair effects of apoptotic cells in a chondroprotective model [34]. Despite these advancements, a deeper understanding of how the physical properties of apoptotic-cell-inspired nanoparticles (e.g., size, shape, and stiffness) influence their ability to modulate macrophage behavior and anti-inflammatory responses is needed [35,36,37]. Based on the previous studies, we demonstrated the efficacy of deformable PS-modified micro-nanomaterials and N-acetylcysteine (NAC) delivery vehicles in suppressing inflammation in an M1 macrophage model for chronic wounds and pulmonary injury, respectively. This work further strengthens the foundation for exploring apoptotic-cell-inspired anti-inflammatory strategies [21,22].

To explore how nanotherapeutics inspired by apoptotic cells target inflammation mediated by macrophages in psoriasis, in this study, we produced two types of PS-conjugated microvesicles with different Young’s moduli, namely C-PSVs and D-PSVs. When co-cultured with LPS- and IFN-γ-stimulated BMDMs in vitro, both C-PSVs and D-PSVs effectively suppressed the inflammatory response and reduced the expression of pro-inflammatory cytokines. Transcriptomic analysis showed similar gene expression profiles, characterized by a marked inhibition of M1 macrophage polarization. The results above indicated that the anti-inflammatory effects of these vesicles were primarily mediated by PS properties rather than Young’s modulus [38].

In addition, our study found that the persistent surface adherence of D-PSVs to macrophages and their resistance to internalization played a critical role in suppressing M1 macrophage polarization. More importantly, this behavior contrasted with the rapid internalization and subsequent lysosomal degradation observed with C-PSVs. The sustained presence of D-PSVs on the macrophage surface effectively blocks continuous LPS and IFN-γ stimulation, mimicking a reduction in stimulus exposure and leading to a more pronounced suppression of M1 polarization and the accumulation of inflammatory factors in the skin microenvironment. This suggests that the physical properties of D-PSVs, such as their deformability and surface adherence, are crucial for their anti-inflammatory efficacy and immune balance restoration.

In the IMQ-induced psoriasiform mouse model, D-PSVs displayed superior therapeutic efficacy compared to C-PSVs. This enhanced effectiveness can be attributed not only to the anti-inflammatory properties previously described but also to the improved skin permeability. Transdermal drug delivery holds promise for treating skin diseases due to its non-invasive nature and avoidance of first-pass metabolism and gastrointestinal complications [39,40]. However, the stratum corneum, the outermost layer of the skin, poses a significant barrier to drug permeation. Benefiting from their intrinsic flexibility and extensibility, D-PSVs efficiently traverse the intercellular spaces of the stratum corneum, facilitating deeper dermal diffusion. The superior transdermal permeation efficiency of D-PSVs compared to C-PSVs can be attributed to their lower Young’s modulus and increased flexibility, characteristics akin to deformable liposomes [41]. These properties enable D-PSVs to undergo deformation, allowing them to navigate through the narrow intercellular spaces within the lipid matrix of the stratum corneum. In contrast, C-PSVs, due to their higher Young’s modulus and lower deformability, face greater challenges in penetrating these restrictive intercellular spaces, resulting in reduced transdermal diffusion. Furthermore, clinical trials investigating D-PSVs in herpes zoster-related skin inflammation demonstrated significant therapeutic outcomes, including reduced redness and swelling, rapid papule reduction, and expedited blister resolution. These results highlight the robust anti-inflammatory capabilities of D-PSVs and their potential as an innovative therapeutic modality for skin inflammatory diseases.

In summary, we developed easily prepared, macrophage-targeted, and phosphatidylserine-exposing microvesicles capable of binding to macrophages and reducing the expression of inflammatory factors. These biomaterials can mitigate skin inflammation in a non-invasive manner by consistently blocking M1 macrophage polarization and further restoring the immune microenvironment. Therefore, our study presents a promising therapeutic strategy for skin inflammatory disorders and holds significant potential for clinical translation.

## 4. Materials and Methods

### 4.1. Agents

Macrophage colony-stimulating factor (M-CSF), interferon-γ (IFN-γ), interleukin 4 (IL-4), and interleukin 13 (IL-13) were obtained from PeproTech Inc. Lipopolysaccharide (LPS), cholesterol, and sodium deoxycholate (SDC) were purchased from Sigma-Aldrich. 1,2-dioleoyl-sn-glycero-3-phosphocholine (DOPC) and 1,2-dipalmitoyl-sn-glycero-3-phospho-L-serine (DPPS) were purchased from Wuhan xin wei Ye chemical Co., Ltd. (Wuhan, China). Antibodies for cell identification, including allophycocyanin (APC)-conjugated anti-mouse CD206, fluorescein (FITC)-conjugated anti-mouse CD86, peridinin-chlorophyll-protein (PerCP)-conjugated anti-mouse F4/80, phycoerythrin (PE)-conjugated anti-mouse CD11b, and antibodies targeting mouse IL-1β, IL-6, inducible nitric oxide synthase (iNOS), IL-10, and arginase 1 (Arg-1), were procured from BD Biosciences (Franklin Lakes, NJ, USA) and BioLegend (San Diego, CA, USA).

### 4.2. Preparation of PSVs

Deformed PSVs (D-PSVs) were fabricated using a thin-film hydration-extrusion method. Briefly, a lipid mixture containing DOPC (6.5 μmol), DPPS (1.5 μmol), and cholesterol (2 μmol) was dissolved in chloroform. The organic solvent was removed through a combination of rotary evaporation and high-vacuum desiccation (70 °C, 2 h) to form a thin lipid film. This film was then hydrated with 1 mL of phosphate-buffered saline (PBS) containing 0.8 μmol of SDC, resulting in a deformable liposome suspension with a total lipid concentration of 10 μmol/mL. Then, this suspension was sonicated using a probe sonicator (180 W, 1 min) for efficient dispersion, followed by extrusion through a 100 nm polycarbonate membrane four times to achieve uniform D-PSVs with a defined size. Conventional PSVs (C-PSVs) were prepared identically, excluding the addition of SDC [23].

### 4.3. Characterization of PSVs

The particle size, polydispersity index (PDI), and zeta potential were determined using a Zetasizer (Mastersizer 3000, Malvern, UK). Transmission electron microscopy (TEM) was employed to visualize the morphology of PSVs. Briefly, a drop of diluted PSV dispersion was adsorbed onto a carbon-coated copper grid (230 mesh). The excess solution was removed using filter paper. The grid was then stained with a 2% sodium phosphotungstate solution for 2 min at room temperature and air-dried. TEM images were acquired using a JEM-1400 electron microscope (JEM-1400 Flash, JEOL Ltd., Tokyo, Japan). Annexin V-FITC (BD Biosciences) was employed to specifically bind to PS to evaluate the extent of PS exposure on the surface of the PSVs, with microvesicles lacking PS serving as a negative control. The labeled samples were analyzed by flow cytometry (BD FACSCanto, BD, Franklin Lakes, NJ, USA). The Young’s modulus was determined using an atomic force microscope (AFM, Bruker Dimension FastScan, Bruker Corporation, Billerica, MA, USA) in PeakForce tapping mode, as previously described [21].

### 4.4. Cells and Animals

Naive bone marrow-derived macrophages (BMDMs) were isolated from the femurs of 8-week-old male C57BL/6 mice. Briefly, after flushing the femurs with cold PBS, the cell suspension was filtered through a 100 μm strainer and centrifuged at 800× *g* rpm for 10 min. The red blood cells were lysed on ice for 10 min. The isolated BMDMs were cultured in DMEM supplemented with 10% fetal bovine serum (FBS), 100 U/mL penicillin, 100 μg/mL streptomycin, and 50 ng/mL M-CSF at a density of 10^6^ cells/mL in 6 cm dishes for 7 days. These macrophages (M0 macrophages) were then stimulated with 100 ng/mL LPS and 20 ng/mL IFN-γ for 24 h to generate pro-inflammatory macrophages (M1 macrophages). Anti-inflammatory macrophages (M2 macrophages) were generated by stimulating M0 macrophages with 20 ng/mL IL-4 for 24 h. Additionally, the RAW 264.7 macrophage cell line was obtained from ATCC and cultured in complete DMEM containing 10% FBS at 37 °C and 5% CO_2_.

Male BALB/c and C57BL/6 mice (postnatal 8 weeks) were purchased from the Animal Center of Southern Medical University and housed under standard conditions at the Animal Center of Jinan University. All animal experiments were conducted according to protocols approved by the Animal Ethics Committee of Jinan University (Approval Code: 20220225-34). For all in vivo procedures, mice were anesthetized with an intraperitoneal injection of 1% pentobarbital sodium (45 mg/kg) to ensure minimal discomfort.

### 4.5. Quantitative Real-Time Polymerase Chain Reaction (qRT-PCR)

For mRNA qRT-PCR, the total RNAs from the cells and tissues were extracted and used as templates for cDNA synthesis. The reverse transcriptase reactions contained 400 ng of total RNA, 4 μL of 5× buffer, 2 μL of 10× nucleic acid mix, 2 μL of reverse transcriptase mixture, and nuclease-free water. The cDNA was diluted 1:3, and 2 μL of the diluted template was used per 20 μL of the qRT-PCR assay. All PCRs were performed using a Bio-Rad CFX Connect Real-Time system (Bio-Rad Laboratories, Hercules, CA, USA), and the data were collected using the Bio-Rad CFX Manager software (version 2.0). The relative expression levels of the targeted mRNAs were normalized against the expression of *β-actin*. The fold changes in the expression between the treatments and controls were calculated by the 2^−ΔΔCt^ method. The efficiency of qRT-PCR performance for target genes is between 100–105%. All data were derived from three different independent experiments. The sequences of the primers employed in the experiments are detailed in Supplementary Table S1.

### 4.6. Transcriptomic Analysis

The M1 macrophages derived from BMDMs were treated with C-PSVs or D-PSVs (0.5 μmol/mL total lipid concentration) for 24 h. Total RNA was extracted using the TRIzol reagent (Invitrogen, Carlsbad, CA, USA) following the manufacturer’s guidelines. The quality of the RNA used for sequencing was assessed using a Bioanalyzer 2100 (Agilent, Santa Clara, CA, USA), and cDNA libraries for each sample were constructed using a Seq-Star™ Rapid RNASeq Kit (Illumina, San Diego, CA, USA). The cDNA samples were sequenced using the Illumina Novaseq 6000 (LC Bio Technology Co., Ltd., Hangzhou, China).

The RNA-seq data were analyzed using bioinformatics tools, with a threshold set at a fold change of ≥2.0 (up- or downregulation). The enrichment analysis of Kyoto Encyclopedia of Genes and Genomes (KEGG) pathways was performed using the differentially expressed genes between the M1 macrophage group and the C-PSV or D-PSV treated groups. These enrichment analyses were performed by METASCAPE (version 3.5, https://metascape.org/gp/index.html#/main/step1/, accessed on 3 January 2025) and DAVID software (Database for Annotation, Visualization and Integrated Discovery, version 6.8, https://davidbioinformatics.nih.gov/, accessed on 3 January 2025).

### 4.7. Treatment of the Imiquimod-Induced (IMQ-Induced) Psoriasis Mouse Model

An IMQ-induced psoriasis mouse model was established in this study [42]. Briefly, the dorsal skin of male BALB/c mice (postnatal 8 weeks) was shaved in a 2 cm × 2 cm area. Subsequently, these mice received daily topical applications of 62.5 mg of Aldara cream containing 3.125 mg of imiquimod (Med-shine Pharma, Chengdu, China) on the shaved area. Drug administration commenced concurrently with the model establishment. For continuous 7-day administration, mice received daily topical applications of 5% IMQ cream at 9:00 a.m., followed by treatment with D-PSVs and C-PSVs at 3:00 p.m. The control group and the IMQ model group received daily applications of 70 μL of saline solution. The high-dose C-PSV group received 70 μL of 10 mM C-PSV solution. The high, medium, and low-dose D-PSV groups received daily applications of 70 μL of 10 mM, 3.3 mM, and 1 mM D-PSV solution, respectively. For alternate-day administration, mice received daily topical applications of 5% imiquimod cream at 9:00 a.m., followed by treatment with 70 μL of 10 mM D-PSV or C-PSV solutions at 3:00 p.m. on days 1, 3, 5, and 7. The control group and the IMQ model group received daily applications of 70 μL of saline solution throughout the study.

**Assessment of psoriatic skin lesions:** The severity of psoriatic skin lesions in mice was evaluated daily using the Psoriasis Area and Severity Index (PASI) score [42]. The PASI incorporates objective measures of erythema, scaling, and induration/plaque thickness to provide a comprehensive assessment of disease severity. Back skin lesions were photographed daily for visual documentation. On days 5 and 7 post-treatment, mice were euthanized, and dorsal skin and major organs were collected for further analysis.

**Histological analysis:** Dorsal skin samples were fixed in 10% formalin, paraffin-embedded, and sectioned at 5 μm thickness. Hematoxylin and eosin (H&E) staining was performed to assess overall tissue morphology using a NanoZoomer S360 (Hamamatsu Photonics K.K., Hamamatsu, Japan). Immunohistochemistry (IHC) was performed to evaluate the expression of specific protein markers, including IL-1β, IL-6, iNOS, IL-10, and Arg-1. The proportion of positive staining was quantified using ImageJ software (Fiji version with Java 6). All data were derived from three different independent experiments.

### 4.8. Permeation and Retention of PSVs

BALB/c mice were depilated one day prior to the experiment. The dorsal area (2 cm × 3 cm) was shaved, followed by the application of a depilatory cream. Residual cream was meticulously removed using a moistened cotton swab. The mice were then separated into D-PSV-treated and C-PSV-treated groups, and 70 μL of DID-labeled D-PSVs or C-PSVs (DID:total lipid molar ratio = 6:1000) were uniformly applied topically to the shaved dorsal area. Mice were then returned to their cages and maintained under subdued lighting. Complete skin samples were collected at 6, 12, and 24 h post-application and immediately imaged using three-dimensional laser confocal microscopy. ImageJ software was employed to quantify the average fluorescence intensity of D-PSVs and C-PSVs in the 3D images acquired at each time point. Additionally, cumulative fluorescence intensity was analyzed. Skin sections were labeled with FITC and scanned using confocal laser scanning microscopy (CLSM) to evaluate the penetration depth. Serial z-stack sections of 100 μm thickness were acquired throughout the tissue. All data were derived from three different independent experiments.

### 4.9. The Cell-Surface Adhesion Analysis of PSVs

RAW264.7 cells (10^5^ cells/well) were seeded onto confocal culture dishes and cultured overnight. Then, the cells were treated with PSVs loaded with fluorescein-DHPE (molar ratio, fluorescein-DHPE:total lipids = 6:1000) at a final lipid concentration of 0.5 μmol/mL in DMEM medium for 3 h. The medium was replaced with fresh DMEM, and the cells were incubated for an additional 3, 6, or 24 h. Washed three times with PBS, the cells were fixed with 4% paraformaldehyde for 15 min. The cells were stained with 0.02 μmol/mL Dil (Invitrogen, Carlsbad, CA, USA) and imaged using a Zeiss LSM 880 with an AiryScan confocal microscope (Zeiss, Oberkochen, Germany) to visualize the cell membranes. All data were derived from three different independent experiments.

### 4.10. The Evaluation of M1-Polarized Suppression by PSVs

BMDMs were isolated, seeded onto 6-well plates (10^6^ cells/well), and incubated for 24 h. The inducing agents were added to the DMEM medium and treated for 12 h to induce M1 polarization. Then, the cells were treated with either C-PSVs or D-PSVs at a final lipid concentration of 0.5 μmol/mL for 24 h. For the cell-surface adhesion assessment, BMDMs were incubated with 0.5 μmol/mL C-PSVs or D-PSVs for 3 h. Following this incubation, the cells were washed thrice with PBS to remove unbound PSVs. Subsequently, the cells were cultured in fresh medium for 9 h before the addition of an M1 inducer for a further 24 h incubation period. BMDMs were incubated with 0.5 μmol/mL of C-PSVs or D-PSVs for 3 h, washed three times with PBS, and then cultured in fresh medium for 12 h. The cells were harvested, treated with an Fc receptor blocking reagent (BioLegend) for 10 min, and incubated with either FITC-conjugated anti-mouse CD86 antibody or APC-conjugated anti-mouse CD206 antibody (BioLegend) for 30 min. Following three washes with PBS, the cells were resuspended in 200 μL of PBS and analyzed immediately by flow cytometry. Data analysis was performed using FlowJo V10 software, with mean fluorescence intensity (MFI) representing the average fluorescence intensity of positive cells. All data were derived from three different independent experiments.

### 4.11. Statistical Analysis

The mice were randomly assigned to treatment or control groups. No animals were excluded from the statistical analysis. Basic pairwise comparisons were performed by independent samples *t*-tests, and a one-way ANOVA was performed for three-group comparisons. Within times-point pairwise assessments of group differences were rendered in terms of 95% confidence intervals to convey effect sizes and their patterns over time. All analyses were performed using Prism (version 7.0; GraphPad, La Jolla, CA, USA) software, and the data are expressed as the mean and standard error of the mean (mean ± S.E.M.).

## Data Availability

The data that support the findings of this study are available from the corresponding authors upon reasonable request.

## Supplementary Materials

The following supporting information can be downloaded at: https://www.mdpi.com/article/10.3390/ijms26010394/s1.

## Abbreviations

AFM	Atomic force microscope
ApoMVs	Apoptotic cell-derived extracellular microvesicles
ATCC	American type culture collection
BMDMs	Bone marrow-derived macrophages
C- and D-PSV	Conventional and deformed apoMVs-inspired PS-containing nanoliposomes
CLSM	Confocal, laser scanning microscopy
DEGs	Differentially expressed genes
DLS	Dynamic light scattering
FITC	Fluorescein isothiocyanate
FBS	Fetal bovine serum
H&E	Hematoxylin and eosin
IHC	Immunohistochemistry
IMQ	Imiquimod
KEGG	Kyoto encyclopedia of genes and genomes
MFI	Mean fluorescence intensity
PASI	Psoriasis area severity index
PDI	Polydispersity index
PS	Phosphatidylserine
SDC	Sodium deoxycholate
TEM	Transmission electron microscopy
