# Supplementary material for: A Low-Modulus Phosphatidylserine-Exposing Microvesicle Alleviates Skin Inflammation via Persistent Blockade of M1 Macrophage Polarization
Source: Int J Mol Sci. 2025 Jan 4;26(1):394. doi: 10.3390/ijms26010394 (PMC11720988; doi:10.3390/ijms26010394)
Supplement: Supplementary file 1 [file ijms-26-00394-s001.zip › Supplementary materials.pdf]

Supplementary Information for

A low-modulus phosphatidylserine-exposing microvesicle alleviates skin inflammation via persistent blockade of M1 macrophage polarization

Zihao Zhang [1,†], Yidi Mo [1,†], Shengxia Xu [1], Lei Jiang [1], Yuanshu Peng [2], Yani ZhuGe [1], Zhijian Su [1], Qi Xiang [1], Rong Zeng [2,*] and Guanglin Zhang [2,3,*]

[1] Guangdong Provincial Key Laboratory of Bioengineering Medicine, Department of Cell Biology, Jinan University, Guangzhou 510632, China; a9343371462022@163.com (Z.Z.); moyidi2022@163.com (Y.M.); xshengx1130@163.com (S.X.); 18279333543@163.com (L.J.);zhugeyani010413@163.com (Y.Z.); tjnuszj@jnu.edu.cn (Z.S.); txiangqi@jnu.edu.cn (Q.X.)

[2] Department of Material Science and Engineering, College of Chemistry and Materials Science, Jinan University, Guangzhou 510632, China; pengys02@163.com

[3] College of Biology and Agriculture, Shaoguan University, Shaoguan 512005, China

* Correspondence: tzengronga@jnu.edu.cn (R.Z.); guanglin.z@sgu.edu.cn (G.Z.)

† These authors contributed equally to this work.

This supplementary file includes:

Tables S1 and S2

Figures S1 to S5

Supplemental Table legends:

Table S1: qRT-PCR primer list

Table S2: The comparison of differentially expressed genes among M0, M1, M2, M1_C and M1_D

Supplemental Figure legends:

Figure S1: Effects of sodium deoxycholate (SDC) on M1 macrophages. M1 macrophages were co-incubated with 20, 40, 80, or 200 μM SDC for 24 h, followed by quantitative PCR analysis of the expression of inflammatory genes. A) TNF-α and B) IL-1β. n = 3.

Figure S2: The illustrates Spearman correlations between global genes and differentially expressed genes (DEGs) in macrophages under various treatments. A) Correlations of global genes and DEGs in M0, M1, M2, and M1 treated with D-PSVs. B) Correlations of global genes and DEGs in M0, M1, M2, and M1 treated with C-PSVs. Each group (M0, M1, M1 with D-PSVs treatment, M1 with C-PSVs treatment) consists of 3 samples, while the M2 group comprises 2 samples.

Figure S3: The morphological observation and the spleen index of psoriasis-like mice treated with C-PSVs and D-PSVs. (A) and (C) Present the morphological depiction of the liver on the 5th day of PSVs treatment in psoriasis-like mice, accompanied by the liver weight-to-body weight ratio. (B) and (D) Exhibit the morphological depiction of the liver on the 7th day of PSVs treatment in psoriasis-like mice, along with the liver weight-to-body weight ratio. C-PSVs-H presents high-dose treatment of C-PSVs. D-PSVs-H, D-PSVs-M, and D-PSVs-L present high-dose, middle-dose and low-dose treatment of D-PSVs, respectively. ns indicates no significant difference; $^{*}P<0.05$, $^{**}P<0.01$, $^{***}P<0.001$.

Figure S4: The qRT-PCR analysis of CD206 gene expression. ns indicates no significant difference. $^{*}P<0.05$, $^{**}P<0.01$, $^{***}P<0.001$.

Figure S5: The sustained inhibitory effect of PSVs on LPS- and IFN-γ-induced M1 macrophage polarization. (-) Represents M1-inducing agents (LPS and IFN-γ) removal from culture medium. ns indicates no significant difference; $^{*}P<0.05$, $^{***}P<0.001$.

Table S1

Gene	Sense	Primer
β-actin	**Forward**	**CTACCTCATGAAGATCCTGACC**
β-actin	**Reverse**	**CACAGCTTCTCTTTGATGTCAC**
Il-1β	**Forward**	**TGCCACCTTTTGACAGTGATG**
Il-1β	**Reverse**	**TGATACTGCCTGCCTGAAGC**
Tnf-α	**Forward**	**GGCAGGTCTACTTTGGAGTCATTGC**
Tnf-α	**Reverse**	**ACATTCGAGGCTCCAGTGAATTCGG**
Il-6	**Forward**	**CTCCCAACAGACCTGTCTATAC**
Il-6	**Reverse**	**CCATTGCACAACTCTTTTCTCA**
Cd206	**Forward**	**CTCTGTTCAGCTATTGGACGC**
Cd206	**Reverse**	**CGGAATTTCTGGGATTCAGCTTC**
Arg-1	**Forward**	**GGAAGACAGCAGAGGAGGTGAA**
Arg-1	**Reverse**	**GGTAGTCAGTCCCTGGCTTATGG**

Figure S1

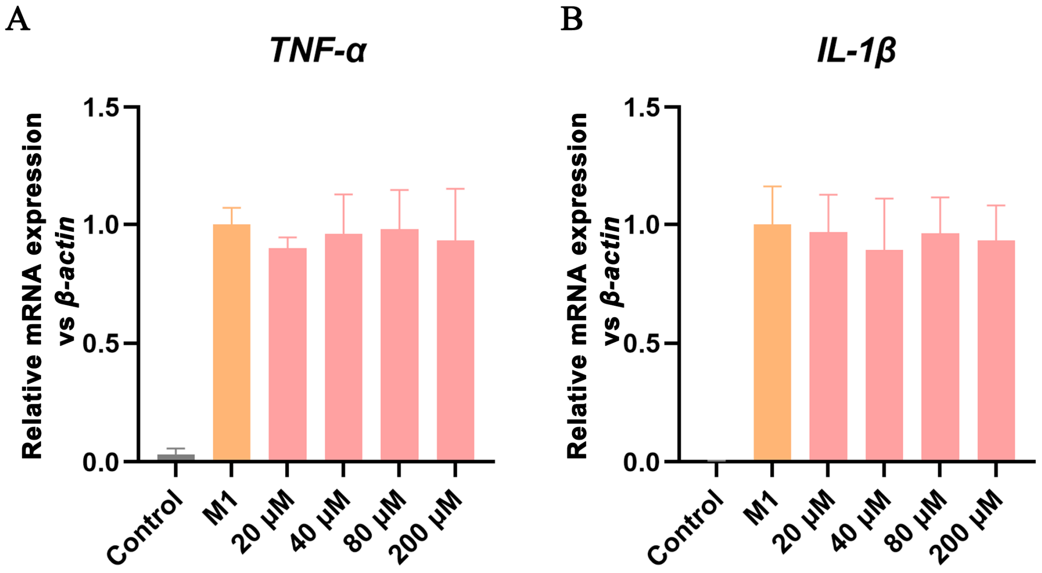
A
TNF-α
Relative mRNA expression vs β-actin
1.5
1.0
0.5
0.0
Control
M1
20 μM
40 μM
80 μM
200 μM
B
IL-1β
Relative mRNA expression vs β-actin
1.5
1.0
0.5
0.0
Control
M1
20 μM
40 μM
80 μM
200 μM

Figure S2

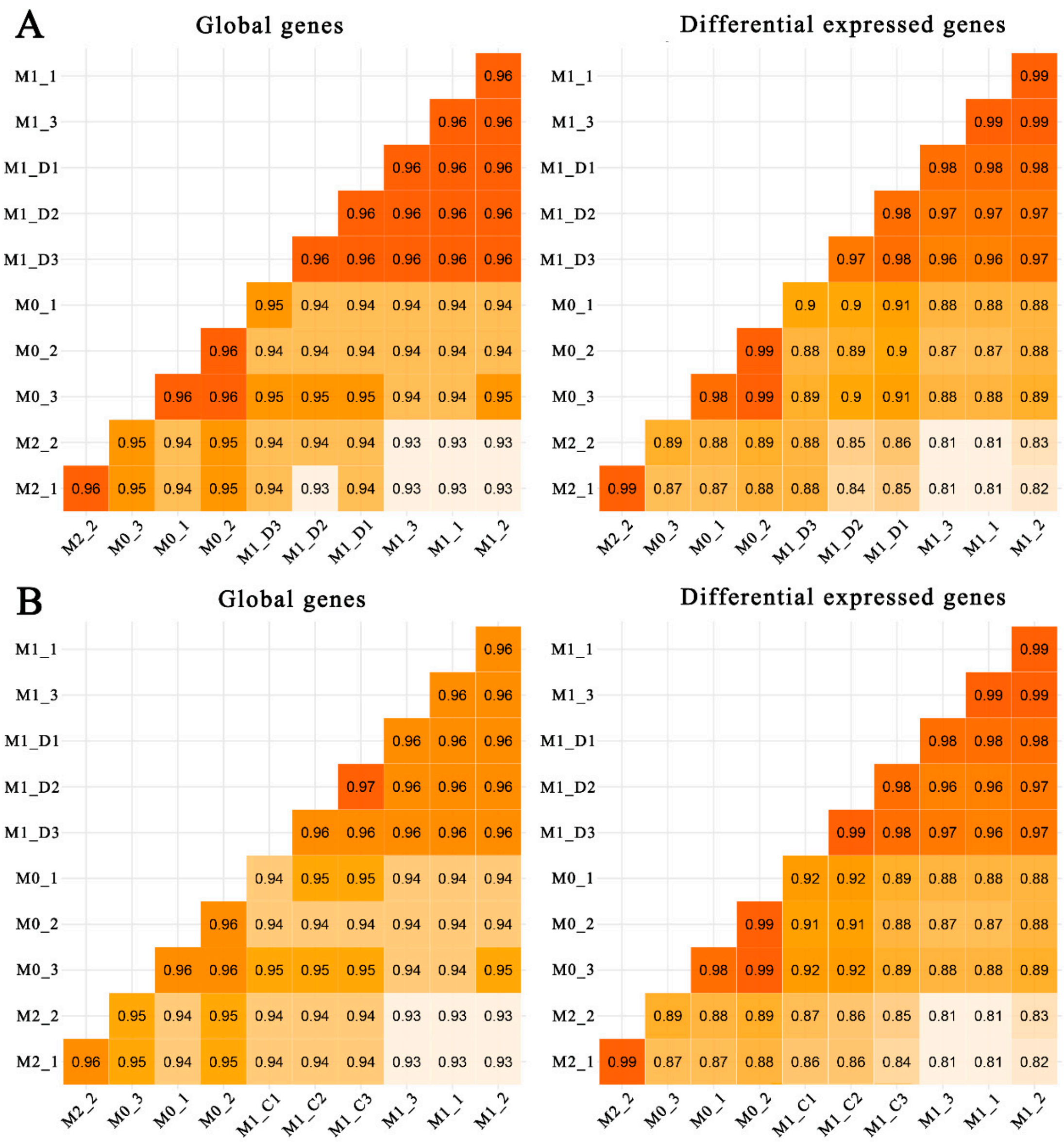
A
Global genes
Differential expressed genes
B
Global genes
Differential expressed genes

Figure S3

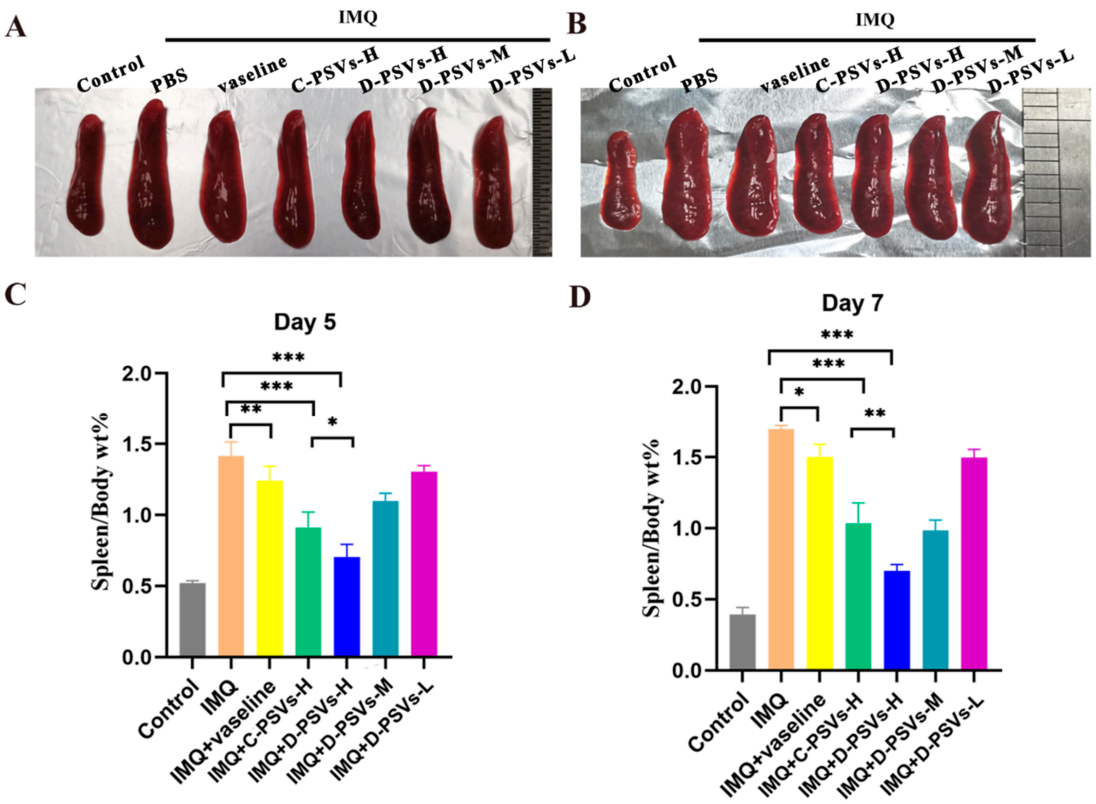

Figure S4

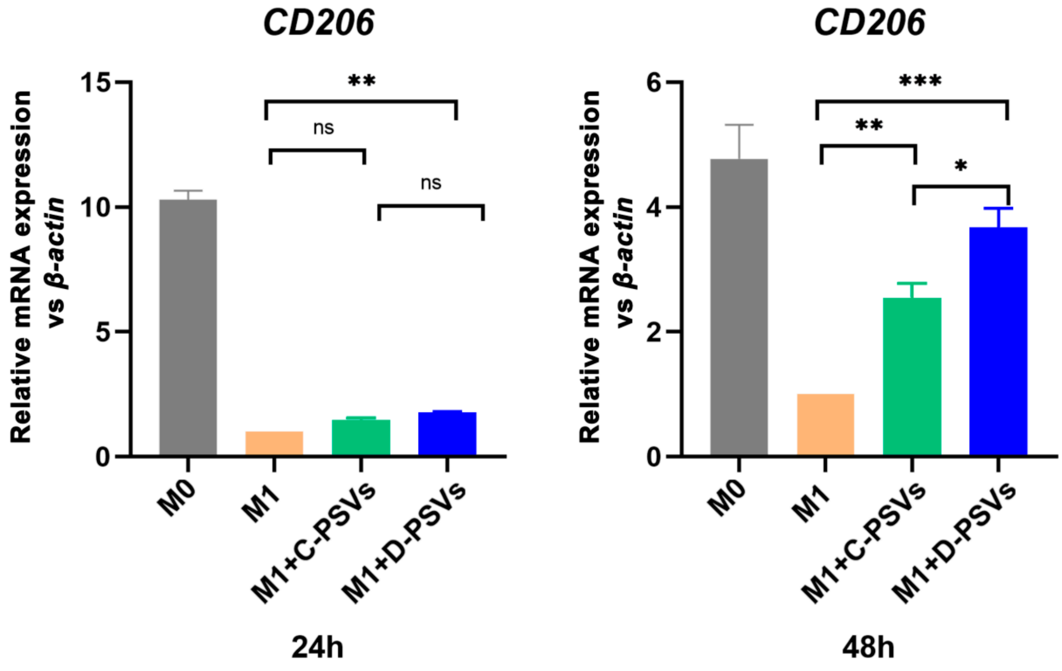

Figure S5

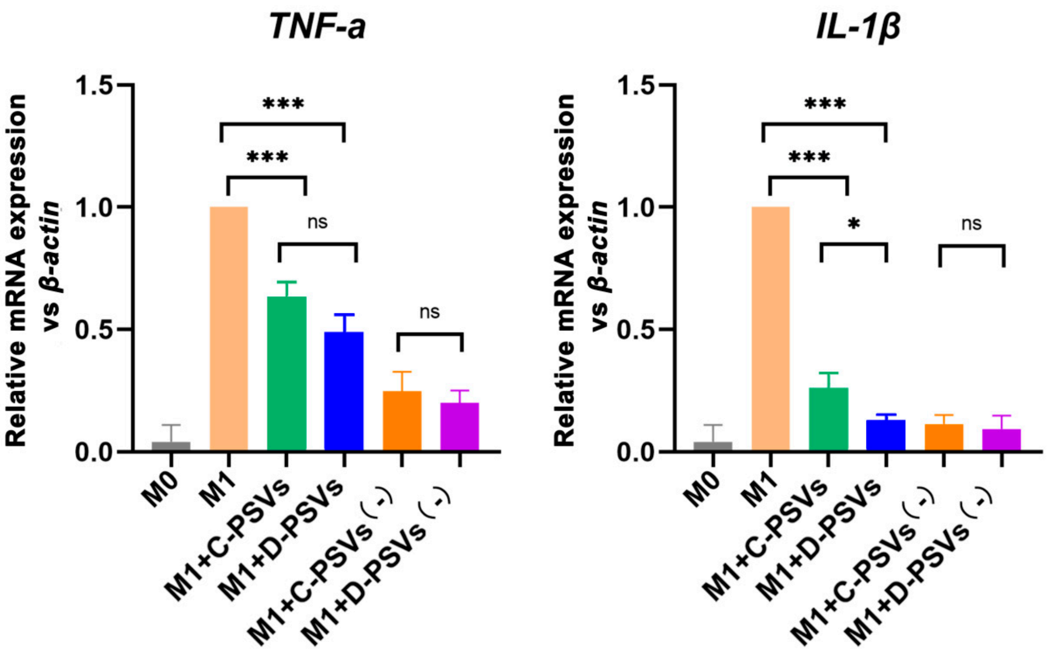

TNF-a
IL-1β
Relative mRNA expression vs β-actin
1.5
1.0
0.5
0.0
***
***
ns
*
M0
M1
M1+C-PSVs
M1+D-PSVs
M1+C-PSVs（-）
M1+D-PSVs（-）
